# Experiences and Perceptions in Dyads about Ostomy Care. Meta-synthesis of Qualitative Studies**

**DOI:** 10.17533/udea.iee.v40n2e12

**Published:** 2022-09-20

**Authors:** Mónica Paola Quemba Mesa, Jenny Katherine Diaz Fernández, Leidy Yemile Vargas Rodríguez, Leila Bautista Plazas, Sandra Patricia Pulido Barragán

**Affiliations:** 1 Nurse, Master’s. Professor, Universidad de Boyacá, Tunja (Colombia). Email: mpquemba@uniboyaca.edu.co. Universidad de Boyacá Universidad de Boyacá Tunja Colombia mpquemba@uniboyaca.edu.co; 2 Nurse, Master’s. Professor, Universidad de Boyacá, Tunja (Colombia). Email: jkdiaz@uniboyaca.edu.co Universidad de Boyacá Universidad de Boyacá Tunja Colombia jkdiaz@uniboyaca.edu.co; 3 Nurse, Master’s. Professor, Universidad de Boyacá, Tunja (Colombia). Email: leiyemvargas@uniboyaca.edu.co Universidad de Boyacá Universidad de Boyacá Tunja Colombia leiyemvargas@uniboyaca.edu.co; 4 Nurse. University Teaching Specialist. Hospital Universitario la Samaritana, Bogotá (Colombia) Email: leylabautistap@hotmail.com Bogotá Colombia leylabautistap@hotmail.com; 5 Nurse. Master’s. Hospital Universitario la Samaritana, Bogotá (Colombia) Email: sandritapuba@gmail.com Hospital Universitario la Samaritana sandritapuba@gmail.com

**Keywords:** nursing, perceptions, patients, ostomy, caregivers, enfermería, percepciones, pacientes, ostomía, cuidadores, enfermagem, percepções, pacientes, ostomia, cuidadores.

## Abstract

**Objective.:**

To understand the experiences and perceptions of care dyads (person and caregiver) when having a permanent discharge ostomy.

**Methods.:**

Meta-synthesis that followed the ENTREQ standards and was registered in PROSPERO CRD42020221755; It was developed in three phases: (i). Search for studies in academic search engines, with MeSH terms: [(Patients) AND (Ostomy)) AND (Caregivers)], including qualitative primary studies published between 2000 and 2020; (ii). Assessment of the methodological quality with the CORE-Q instrument and the credibility of qualitative findings under the guidelines of the Johanna Briggs Institute; and (iii). Comparative analysis according to the guidelines by Sandelowski and Barroso.

**Results.:**

The work identified 664 studies; in screening, 35 passed to full-text analysis; 22 to methodological quality evaluation; and 10 to meta-synthesis. The study found 43 qualitative findings that constituted four categories: dyads perform instrumental and emotional care; ambivalent family caregiving feelings and actions; assertive and effective family care; and rejection of bodily changes and sexual dysfunction. These categories constitute the central meta-theme: “The dyads experience a life rupture, which is restored in a sea of ​ambivalent ​emotions and learning; at the same time, affective, instrumental and assertive care is constructed”.

**Conclusions.:**

People who experience having a permanent discharge ostomy express their rejection to the change in bodily image, alterations in sexual life and as a couple. Caregivers and families are the main source of support by being facilitators in self-care, through relationships of mutuality and reciprocity.

## Resumen

**Objetivo.:**

Comprender las experiencias y percepciones de las díadas del cuidado (persona y cuidador) al tener una ostomía de eliminación.

**Métodos.:**

Meta-síntesis que siguió los estándares ENTREQ y contó con registro en PROSPERO CRD42020221755. Se desarrolló en tres fases: (i) Búsqueda de estudios en buscadores académicos, con términos MeSH: [(Patients) AND (Ostomy)) AND (Caregivers)], incluyendo estudios primarios cualitativos publicados entre los años 2000 al 2020; (ii) Valoración de la calidad metodológica con instrumento CORE-Q y de credibilidad de hallazgos cualitativos bajo lineamientos del JBI; y (iii) Análisis comparativo según los lineamientos los autores Sandelowski y Barroso.

**Resultados.:**

Se identificaron 664 estudios; en el cribado 35 pasaron a análisis de texto completo, 22 a evaluación de calidad metodológica y 10 a meta-síntesis. Se identificaron 43 hallazgos cualitativos que constituyeron 4 categorías: las díadas desarrollan el cuidado instrumental y emocional; sentimientos y acciones de cuidado familiar ambivalentes; cuidado familiar asertivo y eficaz; y rechazo a los cambios corporales y disfunción sexual. Estas categorías constituyen el meta-tema central: “Las díadas viven una ruptura de vida, la cual se restaura en un mar de emociones y aprendizajes ambivalentes; a la vez, se construye un cuidado afectivo, instrumental y asertivo”.

**Conclusión.:**

Las personas que vivencian el tener una ostomía permanente de eliminación, expresan el rechazo al cambio en la imagen corporal, alteraciones en la vida sexual y de pareja. Los cuidadores y las familias se constituyen como la fuente principal de apoyo al ser facilitadores en el autocuidado, por medio de relaciones de mutualidad y reciprocidad.

## Resumo

**Objetivo.:**

Compreender as vivências e percepções das díades de cuidado (pessoa e cuidador) ao ter uma estomia de descarte.

**Métodos.:**

Meta-síntese que seguiu os padrões do ENTREQ e foi registrada no PROSPERO CRD42020221755. Foi desenvolvido em três fases: (i) Busca de estudos em buscadores acadêmicos, com termos MeSH: [(Pacientes) AND (Ostomia)) AND (Cuidadores)], incluindo estudos qualitativos primários publicados entre 2000 e 2020; (ii) Avaliação da qualidade metodológica com o instrumento CORE-Q e credibilidade dos achados qualitativos sob as diretrizes do JBI; e (iii) Análise comparativa segundo as orientações dos autores Sandelowski e Barroso.

**Resultados.:**

664 estudos foram identificados; na triagem, 35 passaram para análise de texto completo, 22 para avaliação da qualidade metodológica e 10 para metassíntese. Foram identificados 43 achados qualitativos que constituíram 4 categorias: as díades desenvolvem o cuidado instrumental e emocional; sentimentos e ações ambivalentes de cuidado familiar; cuidado familiar assertivo e eficaz; e rejeição de alterações corporais e disfunção sexual. Essas categorias constituem o meta-tema central: “As díades vivem uma pausa na vida, que se restabelece em um mar de emoções e aprendizados ambivalentes; Ao mesmo tempo, constrói-se o cuidado afetivo, instrumental e assertivo”.

**Conclusão.:**

As pessoas que vivenciam uma estomia de remoção permanente expressam rejeição à mudança na imagem corporal, alterações na vida sexual e com o parceiro. Os cuidadores e as famílias constituem-se como principal fonte de apoio por serem facilitadores no autocuidado, por meio de relações de mutualidade e reciprocidade.

## Introduction

People who need a discharge ostomy as part of their therapy face bodily changes that impact the roles they represent, also affecting their families and main caregivers, who support them by assuming care activities upon this highly demanding condition; being required to learn to use and access devices for ostomy care and to health services,^1^ which generates economic overload. A long-term adaptation becomes necessary, given that this condition impacts the psychological, social, and sexual dimensions,^2^ along with integral wellness. These characteristics demand additional services from specialized professionals^3^ to promote adaptation and develop self-care because high levels of quality of life are strictly related with high levels of skills for self-care.^4^

Within this context, the informal caregiver is understood as the person with or without a family bond, in charge of making decisions and supporting in daily life activities;^5^ they play a key role in developing the capacity for self-care in patients with ostomy by providing time and effort to offer tangible and emotional care, which is associated with better self-control behavior, better control of the disease, and lower risk of mortality.^6^ People person with permanent discharge ostomy and their informal caregivers develop dyadic affective and care relationships characterized by permanence over time, mutuality, and reciprocity through interpersonal relations.^7^ It is important to understand how these dyadic relationships take place around a challenging health phenomenon, like having a permanent discharge ostomy, given that informal caregivers provide emotional support, support in comprehending the information and in the instrumental care of the ostomy, besides economic support, in food preparation, dressing, in daily life activities, and on reincorporation to the new role; likewise, in the case of older adults or people with limitations to perform their self-care, even more demanding support is required from their informal caregivers. If these relationships are assertive, processes of adaptation to this new health situation are facilitated; however, if there is little or no support, the person with ostomy faces greater difficulties. 

Overall, according with the Federation Association of Incontinence and Ostomy (FAIS), the number of people who currently live with an ostomy in the world is estimated in two-million.^6^ In Colombia, a 2018 study conducted in a health center in Bucaramanga reports the epidemiological profile of the population with ostomies, describing that 57% were men, 51% > 63 years of age, colostomy predominated (71%), and 59.8% of the discharge stomas were temporary.^8^ In turn, these require in their care frequent hygiene and observation of the normal characteristics of the stoma, besides changing their devices.^1^ These care procedures must be learnt by the patient and the caregiver prior to leaving the hospital, but on occasion the patient has upon discharge multiple doubts regarding the instrumental cares and how to confront this new health situation; hence, the dyad faces its situation by diminishing the emotional burden and seeking family support. Due to this, this meta-synthesis seeks to comprehend the experiences and perceptions of the care dyads (person and caregiver) when having a permanent discharge ostomy. 

## Methods

Qualitative meta-synthesis with constant comparative analysis approach by Sandelowski and Barroso specific for meta-synthesis,^9^ under the methodological approach adapted by Arias Murcia (2015),^10^ besides following the standards given by the Enhancing Transparency in Reporting the Synthesis of Qualitative Research (ENTREQ)^11^ with registry in PROSPERO CRD42020221755,^12^. This study was conducted from the qualitative research question: ‘How do care dyads deal with having a permanent discharge ostomy’, which has the following components under the SPIDER structure: Sample: Care dyads conformed by adults 18 years of age and older with permanent ostomy and their informal caregivers; Phenomenon of interest: Carrying a permanent discharge ostomy; Design: Primary qualitative studies (phenomenology, ethnography, grounded theory, among others); Evaluation: Perceptions and experiences of the care dyads; and Research type: Meta-synthesis of qualitative studies.

Three methodological phases were conducted by five researchers (nurses with master's degree, experienced in qualitative research and in caring for people with ostomies). Phase 1 included the search for studies in the databases WOS - Web of Science, PubMed, ProQuest, Academic Search Complete, Clinical Key, Sage, Ovid, Scopus, Google Scholar, and Lilacs, in the Nacional, Sao Paulo, and La Sabana university repositories; in addition to a manual search; using MeSH-validated terms and their equivalents in Spanish and Portuguese *((Patients) AND (Ostomy)) AND (Caregivers)*. The foregoing, by selecting studies that complied with the inclusion criteria of approaching a population constituted by adults 18 years of age and older with permanent ostomy and their informal caregivers, which did not deal with studies in children (this exclusion was contemplated because the relationships of pediatric dyads imply differential characteristics, like increased dependency on care). The work included studies published during the last 20 years (between 2000 and 2020) of qualitative primary designs; the search was carried out from September to December 2020. The identification and selection of the studies was plotted in flow diagram under the PRISMA structure. 

Phase *2* performed the assessment of the methodological quality through the COREQ checklist “Consolidated criteria for reporting qualitative research”^13^ with 32 items organized into three domains: research team and reflexivity, study design and analysis-findings. Each manuscript was evaluated in its full text by two independent reviewers and then in committee to agree on the inclusion of the manuscripts. This process was conducted in Excel spreadsheet when recording the characteristics of the article and the evaluation of the COREQ items (complies, partially complies, or does not comply); 12 studies were discarded due to reporting insufficiently information about the participants, data collection and analysis, and not providing significant findings to the research question. The 10 studies selected complied with at least 50% of the 32 COREQ criteria and passed to the data-analysis phase.

Phase 3, according with Sandelowski and Barroso,^9^ performed the following methodological steps: 1) characterization de studies; 2) extraction of findings, identifying *in-vivo* concepts from each manuscript; 3) grouping of these concepts *in vivo* into common codes defined since their conceptualization and evaluating their level of credibility according to the JBI; for each finding, the following criteria were applied of the different levels of credibility: unequivocal (includes evidence that does not generate doubts, like informed conclusions by the participants and directly observable), credible (includes analyses that are logically inferred in light of the data and the theoretical framework) and not too credible (results not compatible with the data), taking for the study only findings with credible and unequivocal levels.^14^ 4) Identification of similarities and differences of the codes; 5) grouping of codes into thematic categories and definition of their conceptualization; 6) construction of the study’s thematic categories and meta-theme with the definition of their conceptualization. These steps were worked on during weekly meetings for six months with participation by the five researchers, constantly comparing through reading and re-reading of the findings, thus, achieving data maturity. Table 1 displays an example of the analysis process performed for the first category generated, which merges subcategories, identifies their concepts, and highlight their similarities and differences, in such a way that the central categories of analysis generated are structured forcefully. 

**Table 1:** Example of construction of the thematic categories for Category 1 “The dyads develop the instrumental and emotional care of ostomies through a relationship of trust”

Subcategories	Concept	Concept	Similarities	Differences
H4. Learning intention / couple / physical and emotional care	H4. Positive care relationship between couples, which evidences the learning intention to provide physical and emotional support.	Need to learn the instrumental skills of ostomy care to support the partner or person with os-tomy, through trust and bonding they strengthen	Talk about the spe-cific care of the os-tomy and the need to learn to carry it out, to retake daily activities. It manifests the need for support. Identifies positive dynamics in the learning intention	The role of the per-son providing sup-port, sometimes it is the partner, other times it is another family member
H9. Need for support	H9. Need for instrumental support (physical) related with the ostomy upon hospital discharge.			
H46. Adaptation to the new role / instrumental sup-port / acceptance of the changes / independence	H46. Positive care relationship between couples, related with el support in the instrumental care of the person with ostomies; besides, evidences that the person develops independence in self-care			
H47. Trust and bonding / adapta-tion / family affec-tive support / need for companionship	H47. Positive care relationship between couples, related with support in the instrumental care of the person with ostomies; besides, evidences that the person prefers the partner’s company in the self-care			
H48. Trust and bonding / adapta-tion / family affec-tive support / need for companionship	H48. Positive care relationship between couples, related with support in the instrumental care of the person with ostomies; besides, evidences that the person prefers the partner’s company in the self-care			
H27. Trust and bonding / adapta-tion / family affec-tive support	H27. Positive family care relation-ship, with family instrumental support in caring for the person with ostomy			

## Results

**Figure 1:**
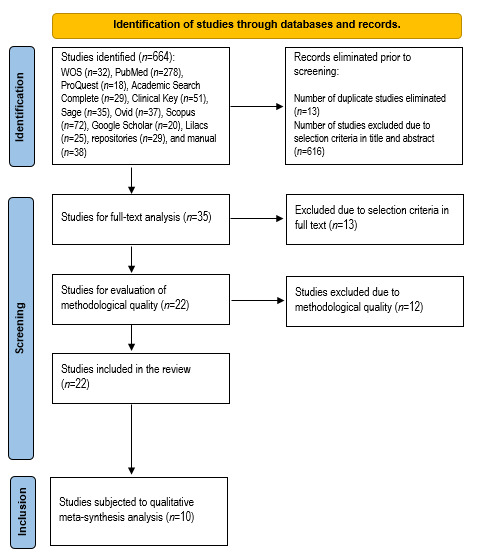
Flow diagram of article selection

The identification and selection of studies is shown in Figure 1; initially, the different databases and search engines identified 664 studies, of these, the work excluded 13 duplicate studies and 616 studies due to not complying with selection criteria upon reviewing titles and abstracts; 35 studies were recovered and reviewed in full text. When applying the selection criteria, 22 passed to the evaluation phase of methodological quality with the COREQ checklist; 12 studies were discarded in this phase, given that these did not comply with 50% of the 32 items proposed in this instrument, with the greatest shortcomings related to the information on personal characteristics and the relation with the participants, data collection and analysis; the studies included had scores ranging between 16 and 29 (Table 2); finally, 10 studies were included in this review and were subjected to qualitative meta-synthesis analysis. 

The studies selected for qualitative meta-synthesis after passing the filters of identification, screening, and selection were published between 2004 and 2020, from six countries, reporting eight in English and two in Portuguese; only three studies used NVivo qualitative data analysis software; most used individual qualitative interviews in their different variables (in depth, semi-structured, telephone, among others) and only one study applied focal groups as part of its methodology; these studies integrated participation from 206 individuals with ostomies and 90 primary caregivers and other relatives (Table 2).

**Table 2:** Characterization of studies selected

Author, year, country and language	Design	Population	Methodological aspects	Methodological quality score	***In-vivo* codes and credibility**
Altschuler *et al*.,^15^ 2018 - USA, English	Qualitative inductive	31 cancer survivors colorectal ostomized and their caregivers. Most of the survivors were 71 years of age or older (67%), women (55%). Two thirds lived with their spouses and received care from them.	In-person interview - semi-structured and demographic questionnaire. Analysis with the NVivo 8 software with inductive analysis techniques.	24.5 / 32	3 *in-vivo* codes; Credibility: 3 credible.
Poletto and Silva, 16 2013 - Brazil, English	Qualitative - Amplified and Shared Clinical Perspective	10 ostomized people and their relatives. No demographic data specified.	Semi-structured interviews. The analysis was through stages according with Trentini and Paim.	21/ 32	2 *in-vivo* codes; Credibility: 2 credible.
Halliday *et al*., 17 2017 - The United Kingdom, English	Qualitative - thematic research	15 ostomized people and 8 caregivers. Median age of patients was 67 years. Most caregivers were spouses or sons/daughters of the patients.	Semi-structured interviews were audio recorded, transcribed textually and anonymized. An inductive thematic analysis was used with the NVivo 10 software.	29 / 32	6 *in-vivo* codes; Credibility: 6 credible.
M^c^Mullen *et al.*,^18^ 2011 - USA, English	Qualitative - ethnographic	31 cancer survivors ostomized and their relatives. Age of patients: 45-70 (32.3%) 71-84 (46.9%), and >85 (19.4%). Relationship with caregiver: spouse (67.7%), son/daughter (6.5%), other relative (19.4%), and not related (6.5%).	In-depth interviews. The inductive theme analysis technique by Strauss was used and qualitative data analysis through the NVIVO8 software	20 / 32	3 *in-vivo* codes; Credibility: 2 credible and 1 unequivocal
M^c^Mullen *et al*.,^19^ 2019 - USA, English	Qualitative - modified grounded theory	57 people with cystectomy and 5 caregivers. Mean age of participating patients was 68 years (range: 38 to 93 years).	Focal groups and in-depth interviews. Analysis performed with focus of modified grounded theory.	20 / 32	2 *in-vivo* codes; Credibility: credible.
Tao-Maruyama,^20^ 2004 - Brazil, Portuguese	Qualitative - ethnographic	12 ostomized people and 5 relatives. Ages of patients range between 45 and 72 years; 7 are women. Caregivers’ ages range between 19 and 71 years, four are women.	Semi-structured interviews in form of narratives and observations of the participants. Interpretation through the interpretive anthropology by Clifford Geertz and by Arthur Kleinman.	25 / 32	14 *in-vivo* codes; Credibility: credible.
Silva and Shimizu,^21^ 2007 - Brazil, Portuguese	Qualitative - life history	10 ostomized people. No demographic data specified.	Semi-structured interviews. The content analysis technique was used	18 / 32	5; Credibility: credible.
Swenne *et al*.,^22^ 2015 - Sweden, English	Qualitative - systematic condensation	19 people in postoperative cytoreductive surgery. Mean age was 56 years (range 32 -79 years).	Individual in-depth telephone interviews. Data were analyzed with systematic text condensation according to the Malterud method.	16 / 32	2 *in-vivo* codes; Credibility: credible.
Sujianto *et al*.,^23^ 2020 - Indonesia, English	Qualitative - phenomenological	10 relatives of people with colostomy. Most were < 40 years of age (60%).	In-depth interviews. Analysis of data in this study used a method created by Colaizz.	16 / 32	1 *in-vivo* code; Credibility: credible.
Villa *et al*.,^24^ 2018 - Italy, English	Qualitative - Interpretative phenomenology	11 people with urostomy. 69 years of age, with age range between 59 and 83. Types of caregivers: family caregiver (8): wife (7) and relative (1), caregiver formal (3).	Semi-structured interviews. Interpretative phenomenological analysis	17.5 / 32	5 *in-vivo* codes; Credibility: credible.
Total	10 studies	206 ostomized people. 90 caregivers and relatives			43 *in-vivo* codes: 42 with credible and 1 unequivocal.

**Figure 2:**
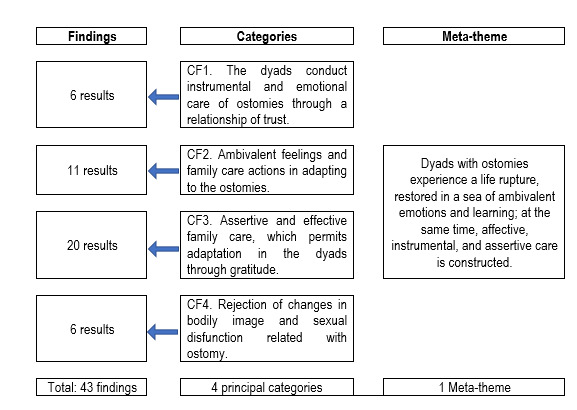
Results of the meta-synthesis of the findings

The work identified 43 findings / *in-vivo* codes, of which upon evaluating their level of credibility - according to the JBI^13^, 42 were credible and one unequivocal (Table 2), which validated their inclusion in this analysis. As shown in Figure 2, four principal thematic categories were structured that constitute the meta-theme that answers the study’s research question: the dyads with ostomies experience a life rupture, restored in a sea of ambivalent emotions and learning; at the same time, affective, instrumental, and assertive care is constructed. The categories emerging from the analysis centered around four principal ones, which mention performance of instrumental activities in direct care of the ostomy and progressive acquisition of trust when overcoming the fear of making mistakes. Another fundamental aspect is the ambivalence in the feelings and care actions experienced by the dyad, where they go through moments of fulfillment, acceptance, and trust; but also, through moments of crisis, feeling of abandonment, grief or loss of identity and quality of life. Moreover, it is perceived that assertive care and companionship from the dyads generate satisfaction, feelings of appreciation and a bond of security. Lastly, as indicated in the fourth category, rejection of the new bodily image, loss of their self-esteem and negative self-perception, after the creation of the discharge stoma, as well as loss of sexual functionality sexual when establishing relationships secondary to the perception of rejection or of having accidents with the collection devices.

**Figure 3:**
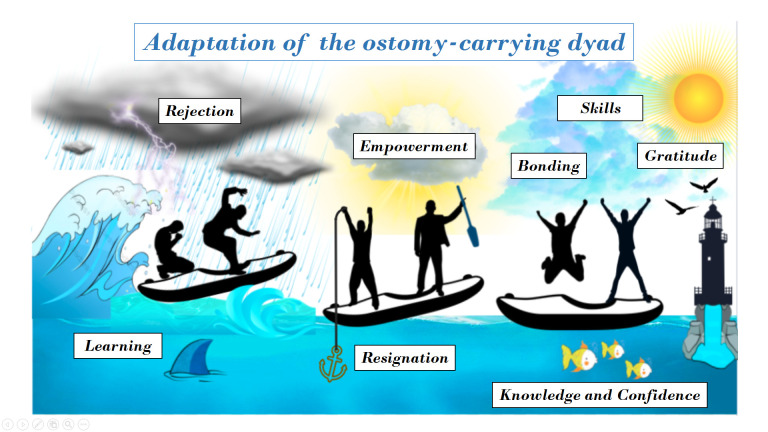
Scheme of the meta-theme. Source: by the authors

In the synthesis of perceptions and experiences of people with a permanent discharge ostomy and their informal caregiver, through the metaphor of a sea of emotions that integrates the resulting meta-theme (Figure 3), which - as of the literature consulted - sought to interpret the different stages the dyad must go through to achieve the new normality. This includes an initial moment characterized by chaos and imbalance, where the ostomy originates and the dyad must carry out instrumental learning on the principal care, likewise, it must gain knowledge on the diet and necessary clothing to maintain quality of life. At this point, the ostomized person requires psychological, emotional, economic, and social support to continue with the challenges of the new role. Some individuals may receive this support, which aids in the adaptation process, but - in contrast - some people receive little or no support, which causes greater stress and difficulty to reach acceptance of the new reality. Thereafter, comes a stage that again seeks equilibrium within that turbulent sea, where a higher level is reached in achieving competencies on instrumental knowledge and skills, as well as ambivalent feelings about carrying an ostomy, where positive and negative feelings are experienced in the dyad to accept the new dynamics of life with the permanent discharge stoma in external environments, like the labor, social, academic, dynamics and in recreational settings, as well as in the intimate setting, like the perception of self-image, self-esteem, the relationship a partner and sexuality, reaching as maximum goal empowerment and acceptance of the new reality through acceptance or from spiritual support strengthened by faith and resignation. Finally, in the last stage where a peaceful sea is presented; complete acquisition of instrumental skills is achieved and knowledge is perceived with greater confidence; the dyad expresses dominance in the daily activities of ostomy care and reaches a bond based on gratitude and on the assertive relationship.

### Adaptation of the ostomy-carrying dyad

The following categories identify the interpretation of the findings and *in-vivo* codes that structured four principal thematic categories:

### Category 1. Capacity of the dyads to perform instrumental and affective/emotional care of the ostomies mediated by a relationship of trust and bonding

This category shows a positive relationship of the dyads where their partners and caregivers display that learning intention to support not only physical but emotional care, allowing individuals to develop independence in their self-care; this is given in the following dialogues:

*Altschuler et al.,*^15^ *pg. 533: ... ¿(your husband) does he refuse to help with your ostomy?*

P: *No, in fact, he says he wants to help me. He wants to be a caregiver. He’s learning very slowly and I guess I hope that by the time I really need help, he'll have trained well enough to not only do the physical stuff, but be there for me emotionally.*

*Villa et al.,* 24 *pg. 49: It was difficult at the beginning. My wife helped me change two or three times, but then I started doing it on my own and now I am increasingly faster.*

*Villa et al.,* 24 *pg. 50: Then I have to change the bag when it is almost full and the plate every other day. I can do it on my own, but I prefer if it’s both of us doing it... my wife helps me.*

### Category 2. Ambivalent feelings and family care actions in adapting to the ostomies

Relationships of care are characterized by moments where contrasting emotions exist, given that family support is manifested with episodes of fear, resignation, frustration, denial in the dyad, exhaustion and tiredness by the caregiver; this within a context in which care is seen as demanding human and economic resources. The relationships of the dyads are ambivalent in that family support is manifested during its initial phases in processes that fluctuate between fear and grief. Over time, acceptance of the disease is achieved, as well as the search for support and acquisition of abilities to adapt to the new life reality. The dialogues evidence the initial processes of rejection and difficulty in accepting the process of living with an ostomy:

*Poletto and Silva,* 16 *pg. 535: It was a nurse who did the cleaning and showed my daughter how the cleaning would be done. There, she did it near my daughter and showed how is was done, so it works... So, it's one thing that leaves us scared from the start. It is something new that you know nothing about, you don’t know the procedure…*

*McMullen et al.,* 19 *pg. 58: My wife said: No, I love you, but I will not do that [tubes for washing and changing bandages].*

Further, the dialogues show resignation as part of acceptance and adaptation:

*Halliday et al.,* 17 *pg. 5: Yeah, well, it's, I mean, as much as you've been sorry about it sometimes: that damn thing. You know, that's been your lifeline.*

*Tao-Maruyama,* 20 *pg. 135: For her, the colostomy is a necessity she had. She had no other solution for this… Today, I am used to it, I never touched it or asked about it because I know she strays from it a bit.*

Finally, and after some time, people with ostomies manifest adaptation to living with and caring for their ostomy; some have support from their partners or families; they even mention processes of overprotection that reduce their independence.

*Silva and Shimizu,* 21 *pg. 309: I have much support from my family. I am very spoiled. Sometimes I even say, people, I'm not dead... (Rita).*

*Silva and Shimizu,* 21 *pg. 310: My kids don't want me to do anything, but I don't have the nature to sit still. I grew up on the farm with a heavy work and if I shut up, I think it's bad.*

### Category 3. Assertive and effective family care, which permits adaptation in the dyads and expressed through gratitude

The dyads of person with ostomy and family caregiver evidenced adaptation processes, characterized because positive coping is assumed from the new condition that allows individuals to rejoin their daily life and project themselves with a future life expectancy; in some dyads and families, the spiritual dimension represents strength in these adaptation processes. The importance of family support for the person with ostomy is highlighted in terms of generating motivation, companionship, an environment that facilitates acceptance, self-esteem, and adaptation to the changes caused by having an ostomy; given by gratitude, bonding, trust, recognition, and empowerment. The dialogues describe positive adaptation processes, more advanced in caring for the physical integrity of the skin, companionship and gratitude, emotional support, and motivation:

*Halliday et al.,* 17 *pg. 5: I'm thankful that the girls are young and totally motivated, you know, to help me. If I were alone, I would find it very difficult, quite difficult... (Female patient)*

*Tao-Maruyama,* 20 *pg. 250: I did not move until my son said: Mom, stop crying, stop being scared, you will be well, you can change the bag! and there I started. Now I change it and clean myself!*

A specific dialogue identifies that feelings of fear are hidden, to show courage in front of their children; like the following:

*Altschuler et al.,* 15 *pg. 50: I wanted to act like a heroine, I wanted to pretend that it was nothing, I wanted to pretend to be a woman who was not afraid of anything, to encourage myself. But I was terribly scared. But, since I knew that my children were waiting for me upstairs, this encouraged me.*

Other dialogues show spirituality and identify it as part of the positive adaptation processes:

*Tao-Maruyama,* 20 *pg. 204: We ask God to give us strength. He is the only one who can give us strength, to endure all the problems.*

### Category 4. Rejection of changes in bodily image vs. sexual problems along with sexual disfunction related with the ostomy

People with ostomies describe loss of integrity in bodily image, which may - in some cases - affect the couple’s relationship, their sex life, and lead to its disfunction. It should be noted that for the person with an ostomy, their corporeality is fundamental, thus, they experience much anxiety and concern regarding their sexual pattern:

*Tao-Maruyama,*^20^ *pg. 234: The only thing I still feel, still fear, is to have an intimate relationship with my husband. He does not accept to see me whole […]*

*Tao-Maruyama,*^20^ *pg. 122: He (husband) had never seen my colostomy until today. Never, ever (...). Until today, he does not accept this change in my body […].*

Some people are deeply affected by their pathology, which is why they would like to forget their situation and perceive the stoma as an indicator to remember it: 

*Swenne et al,*^22^ *pg. 195: I have not initiated any intimate relationship with my spouse. I can’t imagine how I would manage with the stoma. There are so many barriers I must overcome... If I had not had it (the stoma)... it would have been possible to forget all this damn disease as soon as I started to improve, but now I can never do so.*

## Discussion

The meta-syntheses are novel designs that comprised evidence of primary qualitative studies to construct, describe and/or explain phenomenon of interest in research.^25^,^26^ The validity of this methodology is given - among other aspects - by the evaluation of quality of the studies, *in-vivo* identification of concepts, construction of descriptive categories and the meta-theme. With regard to the evaluation of quality of the individual studies, it permits critical reading of the objective, sampling, collection and analysis of information;^27^ for such, some of the tools that contain the minimum standards of qualitative studies are: Consensus-based Checklist for Reporting of Survey Studies (CROSS);^26^ improving reporting of Meta-Ethnography (eMERGe);^28^ qualitative research review guidelines (RATS);^29^ and Consolidated criteria for reporting qualitative research (COREQ).^30^ For this study, COREQ was the tool selected, given that it establishes essential aspects and transversal to the distinct types of qualitative studies. Overall, this methodology represents a more-comprehensive interpretation of phenomena, through constant comparison and integration of the findings; its development allows broadening the relevance and usefulness of qualitative studies.^26^

In relation to the results of this qualitative meta-synthesis, as core elements of the meta-theme, it is highlighted that these dyads experience a life rupture, restored through affective, instrumental, and assertive care. In this regard, studies with similar methodology have identified related findings; highlighting the study by Capilla-*Díaz et al.,*^31^ which conducted a qualitative systematic review also under the Sandelowski theory and evidenced that the experiences of people with ostomy are explained under acceptance, adaptation, and autonomy; these aspects are complemented in that the present study demonstrates that this acceptance and adaptation is facilitated with existence of Assertive and Effective Family Care. 

In turn, Pape E *et al*.,^32^ through a thematic-type synthesis review evidenced from the experiences of the patients how care must focus on management strategies and emotional support; these findings are complemented with those disposed from the first analysis category in the present study that identifies that the dyads conduct instrumental and emotional care of the ostomies through a relationship of trust; within this context, by this being a complex process, it becomes absolutely necessary to involve spouses and close relatives,^33^ with an approach toward the psychosocial aspects of the creation of the ostomy that extends to tangible support to spouses and relatives from the health sector, making it necessary to carry out further research centered on the spouses or relatives 34 in relation to how these dynamic relationships of mutuality and support take place. 

Similarly, the different studies analyzed show people who experience having a permanent discharge ostomy express rejection to change in bodily image due to a rupture in their physical integrity related with the ostomy; likewise, alteration in the sex life and the couple’s relationship is noted - leaving as a result, sexual problems along with sexual disfunction, as confirmed by García and González in their respective 2020 studies.^35^^-^36 It was possible to see some behaviors between the spousal dyads from their sexual needs, such as: shame, fear, discomfort, and rejection due to the loss of their physical dignity.^35^

Among the study findings, it is considered that women are the most affected in their relationship; it is much more difficult to accept or cope with changes in bodily image; their sex life is altered, causing a disfunction of such, related with that of carrying a permanent discharge digestive ostomy.^35^,^36^ Due to this, future research should to delve on the male gender and his dyad, as well as on his perceptions from the sexual perspective related with the ostomy.^35^ Thus, ostomized people and their dyads have sex problems after surgery;^35^,^36^ sexuality is an important aspect in the spousal dyads, besides being closely linked to the bodily image.^36^^-^37 However, the ways of life and culture of each dyad can make a difference with respect to how to cope with the new situation in their lives.^35^

Further, regarding the category of Assertive and Effective Family Care, which permits adaptation in the dyads and is expressed through gratitude; the finding coincide with the care needs of the evident life change and role that impacts upon the dyad with ostomy, in multiple and complex factors related with physical, physiological, mental, and emotional aspects in which - to ensure positive adaptation, these must be satisfied first by the caregiver and secondly from the integral service by the health staff.^37^ Also, Araujo *et al*.,^38^ mention in their study to keep in mind the family as principal element in adapting to the stoma, being that it is in charge of providing emotional and social support to confront problems upon this new situation and achieve adequate self-care and autonomy of the subject. Lastly, it is necessary to corroborate that the power of adaptation has a component from the subjectivity of each member of the dyad, having as starting point circumstances of biographic situation, body of knowledge, and prior experiences.^39^,^40^

With respect to the category of the Capacity of the Dyads to Perform the Instrumental and Affective Care of the Ostomies mediated by a relationship of trust and bonding, as mentioned by Villa *et al*.,^25^ people consider the family the principal source of support; it is facilitating at the beginning, but the ideal is for patients to assume their self-care.^41^ Due to the foregoing, follow-up and health education processes must be carried out in personalized manner, from specific learning needs as indicator of successful management of the recovery after the hospital discharge.^42^

To conclude, the studies analyzed indicate that people who experience having a permanent discharge ostomy and their caregivers undergo a life rupture, which is restored in a sea of ambivalent emotions and learning; while constructing affective, instrumental, and assertive care, as reflected in the meta-theme. Likewise, caregivers and the families become the principal source of support by being facilitators in self-care, through relationships of mutuality and reciprocity. As direct implication for the transdisciplinary clinical practice is the need to assess the individual needs of each care dyad, not merely on instrumental themes and health education, but upon perceiving it integrally, also considering the physical-emotional support and companionship they require; in this sense, these care dyads need to have from health systems formal support and follow-up strategies, including things as important as guaranteeing the necessary supplies. 

Regarding the nursing practice, the importance is highlighted of enhancing academic curricula with thematic contents related with this research phenomenon and the literature evidences the need to strengthen education for people with ostomies and their caregivers, given that when leaving the hospital, they must deal with the reality of assuming care independently and can experience initially feelings of fear and distrust related with not knowing about self-care activities and practices. Moreover, for the different health service provider institutions, it would be recommended to implement support groups to ostomized individuals that include therapeutic approach from different professions, like medicine, psychology, social work, nursing, nutrition and dietetics that offer tools to the dyads and guarantee monitoring to evaluate the capacity to adapt to new changes and roles.

As limitations to this study, it can be recognized that few qualitative studies exist on the theme that - in turn - consider the perceptions of people with permanent discharge ostomies and of their informal caregivers; likewise, many of the primary qualitative studies analyzed do not comply with the minimum methodological parameters provided by the COREQ checklist, given that frequent methodological shortcomings were found in the reports regarding insufficient information on the participants, data collection and analysis, and not contributing significant findings to the research question, aspects that suggest the need to strengthen qualitative studies on this phenomenon in terms of its quantity and quality; besides, the foregoing permits reflecting upon the need to enhance skills in qualitative health research in different academic scenarios.
